# Supplementary material for: Iterative improvement in the automatic modular design of robot swarms
Source: PeerJ Comput Sci. 2020 Dec 7;6:e322. doi: 10.7717/peerj-cs.322 (PMC7924708; doi:10.7717/peerj-cs.322)
Supplement: Supplemental Information 3 [file peerj-cs-06-322-s003.zip › argos3/doc/api/standalone/a00447_source.html]

ARGoS: plugins/robots/foot-bot/control\_interface/ci\_footbot\_motor\_ground\_sensor.h Source File


- Main Page
- Related Pages
- Namespaces
- Classes
- Files

- File List
- File Members

# plugins/robots/foot-bot/control\_interface/ci\_footbot\_motor\_ground\_sensor.h

Go to the documentation of this file.

```
00001 
00032 #ifndef CCI_FOOTBOT_MOTOR_GROUND_SENSOR_H
00033 #define CCI_FOOTBOT_MOTOR_GROUND_SENSOR_H
00034 
00035 namespace argos {
00036    class CCI_FootBotMotorGroundSensor;
00037 }
00038 
00039 #include <argos3/core/control_interface/ci_sensor.h>
00040 #include <argos3/core/utility/math/vector2.h>
00041 #include <argos3/core/utility/math/range.h>
00042 #include <vector>
00043 
00044 namespace argos {
00045 
00046    class CCI_FootBotMotorGroundSensor : public CCI_Sensor {
00047 
00048    public:
00049 
00050       struct SReading {
00051          Real Value;
00052          CVector2 Offset;
00053          
00054          SReading() :
00055             Value(0.0f) {}
00056          
00057          SReading(Real f_value,
00058                   const CVector2& c_offset) :
00059             Value(f_value),
00060             Offset(c_offset) {}
00061       };
00062 
00063       typedef std::vector<SReading> TReadings;
00064 
00065    public:
00066 
00067       CCI_FootBotMotorGroundSensor();
00068 
00069       virtual ~CCI_FootBotMotorGroundSensor() {}
00070 
00071       const TReadings& GetReadings() const;
00072 
00073 #ifdef ARGOS_WITH_LUA
00074       virtual void CreateLuaState(lua_State* pt_lua_state);
00075 
00076       virtual void ReadingsToLuaState(lua_State* pt_lua_state);
00077 #endif
00078 
00079    protected:
00080 
00081       TReadings m_tReadings;
00082 
00083    };
00084 
00085    std::ostream& operator<<(std::ostream& c_os, const CCI_FootBotMotorGroundSensor::SReading& s_reading);
00086    std::ostream& operator<<(std::ostream& c_os, const CCI_FootBotMotorGroundSensor::TReadings& t_readings);
00087 
00088 }
00089 
00090 #endif
```

---

Generated on 10 Jul 2018 for ARGoS by 
 1.6.1
